# Detection of the Early Sensitive Stage and Natural Resistance of Broad Bean (*Vicia faba* L.) Against Black Bean and Cowpea Aphids

**DOI:** 10.3390/insects16080817

**Published:** 2025-08-07

**Authors:** Fouad Meradsi, Adel Lekbir, Oussama A. Bensaci, Abdelkader Tifferent, Asim Abbasi, Assia Djemoui, Nazih Y. Rebouh, Abeer Hashem, Graciela Dolores Avila-Quezada, Khalid F. Almutairi, Elsayed Fathi Abd_Allah

**Affiliations:** 1 Department of Ecology and Environment, Faculty of Natural and Life Sciences, Batna 2 University, Batna 05000, Algeria; 2 Laboratory of Improvement of the Phytosanitary Protection Techniques in Mountainous Agrosystems (LATPPAM), Agronomy Department, Institute of Veterinary and Agricultural Sciences, University Batna 1-Hadj Lakhdar, Batna 05000, Algeria; 3 Food Science Laboratory (LSA), Department of Food Engineering, Institute of and Veterinary Agriculture Sciences, University Batna 1-Hadj Lakhdar, Batna 05000, Algeria; 4 Agronomy Department, Institute of Veterinary and Agricultural Sciences, Batna 1 University, Batna 05000, Algeria; 5 Department of Entomology, University of Agriculture, Faisalabad 38040, Pakistan; asimuaf95@gmail.com; 6 Department of Environmental Management, Institute of Environmental Engineering, RUDN University, 6 Miklukho-Maklaya St., Moscow 117198, Russia; 7 Department of Botany and Microbiology, College of Science, King Saud University, P.O. Box 2455, Riyadh 11451, Saudi Arabia; 8 Facultad de Ciencias Agrotecnológicas, Universidad Autónoma de Chihuahua, Chihuahua 31350, Chihuahua, Mexico; 9 Department of Plant Production, College of Food Science and Agriculture, King Saud University, P.O. Box 2460, Riyadh 11451, Saudi Arabia

**Keywords:** *Aphis craccivora*, *Aphis fabae*, antibiosis, antixenosis, beans, biotic stress

## Abstract

Broad bean is one of the most important leguminous crops worldwide. However, its productivity is greatly affected by the infestation of *Aphis fabae* and *Aphis craccivora* (Hemiptera: Aphididae). The main objective of the current study was to identify the most susceptible phenological stages of the broad bean variety (Histal) against black aphids’ herbivory. This had been achieved through an evaluation of plant resistance mechanisms such as antixenosis and antibiosis. The results regarding an antixenosis test revealed that the four tested phenological stages of *V. faba* did not have a significant effect on the preference of *A. craccivora* and *A. fabae* towards the crop plant. Overall, a slightly higher number of adults settled on the three and four unfolded leaves’ stage of the crop plant. Similarly, the highest number of developed embryos were found in the four leaves’ stage of the crop, and the lowest in the second leaf stage. The adult body size of *A. craccivora* was slightly larger in the case of the three unfolded leaves. Furthermore, the maximum body size of *A. fabae* adults was recorded in the case of the first unfolded leaf stage crop. Linear correlations between the biological parameters for both species revealed only one significant relationship between developed and total embryos for *A. craccivora*. The results of the current study highlight the need to protect broad bean crops against infestations of black aphids, i.e., *A. craccivora* and *A. fabae.* This is essential for reducing direct damage and preventing the transmission of phytoviruses. However, future studies should aim to evaluate the susceptibility of all developmental phenological stages of the crop against black aphids to mitigate potential crop losses.

## Simple Summary

The black aphids are considered the most important insect pest of the broad bean crop, causing damage either directly through sap feeding and indirectly through virus transmission. The objective of the current study was to find out the most susceptible stage of Histal broad bean variety against black aphids. Overall, the results showed that later leaf stages of the crop were more susceptible to black bean aphids than early leaf stages. This study clearly highlights the importance of protecting the most sensitive stage of the bean crop against black bean aphid attacks, in order to minimize crop damage and the transmission of phytoviruses.

## 1. Introduction

Broad bean (*Vicia faba* L., Fabales: Fabaceae) is globally recognized as one of the major large-seeded legume crops, valued for its nutritional composition, culinary versatility, and agronomic benefits [1]. Broad bean fixes atmospheric nitrogen via root symbiosis and provides soil with natural green manures, which significantly enhances the crop yields of successive crops. The crop also diversifies cropping systems, hinders insect pests and diseases, and promotes pollinators by providing them with floral resources [1,2,3]. Broad beans also play a crucial role in promoting food security by providing high protein content, essential vitamins, and mineral nutrients for both human and animal consumption. Moreover, the seeds produced by broad bean are enriched with starch and numerous proteins, which are greatly valued in different food and feed markets, all over the globe [2,3].

Nevertheless, the productivity of *V. faba* is greatly affected by a number of biotic and abiotic stresses which attack the crop plant during its different phenological stages [4,5]. Among different biotic stressors, various fungal diseases, weeds, parasitic plants such as broomrape, and insect pests are of prime importance, which severely affect crop growth and productivity [6,7,8,9,10,11]. The insect pests most frequently recognized for damaging broad beans include black aphids, specifically *Aphis fabae* [12,13,14,15] and *A. craccivora* [16]. Moreover, *Bruchus rufimanus* [17] and *Sitona lineatus* also affect crop growth at various plant stages [18,19,20].

Among broad bean insect pests, black aphids are considered most notorious maily due to their diverse reproduction methods, complex life cycle, close association with their hosts, and polyphenism [21,22,23]. Among black aphids, *Aphis fabae* (black bean aphid) and *A. craccivora* (cowpea aphid) were among the 14-aphid species which cause greatest damage to different agricultural crops, including broad beans. Moreover, the legume family is ranked as the 9th largest plant family in terms of aphid herbivory [23].

The black bean aphids, *A. fabae* and *A. craccivora,* are sap-sucking insect pests which prefer newly grown plant tips to deposit its nymphs. The feeding of both insects is responsible for disrupted plant growth, leaf distortion, phytotoxicity, and honeydew secretion, leading to a potential reduction in photosynthesis Moreover, sap sucking from inflorescence, tender shoots, and pods results in the drying up of tender shoot and the premature fall of flowers, flower buds, and tender pods [21,22]. Moreover, *A. fabae* is a highly polyphagous pest, with a host range encompassing over 200 plant species worldwide [23]. It can transmit more than 30 plant viruses, including non-persistent viruses affecting beans, peas, beets, crucifers, cucurbits, dahlia, potato, tobacco, tomato, and tulip. Additionally, it can also transmit the persistent Beet Yellow Net Virus (BYNV) and Potato Leaf Roll Virus (PLRV) [23]. Similarly, *A. craccivora* also has a wide host range within many plant families, including the Fabaceae such as *Arachis* spp., *Colutea* spp., *Glycine* spp., *Medicago* spp., *Melilotus* spp., *Trifolium* spp., and *Vicia* spp. It attacks around 50 crops from 19 different plant families and is involved in the spread of about 30 plant viruses. These include non-persistent viruses affecting beans, cardamom, groundnuts, peas, beets, cucurbits, and crucifers, as well as the persistent Subterranean clover stunt virus and Peanut mottle virus [23].

The excessive and irrational use of synthetic insecticides, combined with their high application costs, often leads to environmental contamination, harmful effects on insect natural enemies including insect pollinators, and the emergence of resistant insect biotypes. These beneficial insects (natural enemies and pollinators) are crucial not only for the biological control of insect pests but also for the maintenance of natural equilibrium of ecosystems. Additionally, insecticide residues can persist in soils and plant tissues, such as bean pods, which can subsequently pose numerous risks to human health [24,25,26].

Host plant resistance is a vital component of different pest management programs, often acknowledged for its ecological and economic benefits [27]. Plants usually use antibiosis and antixenosis mechanisms to avoid external intruders. Antibiosis is a natural resistance mechanism, which negatively affects the biology or physiology of the arthropod pest; the impact usually translates into reduced insect survival, growth, and reproduction [28,29]. However, in the case of antixenosis, the resistant plant exhibits characters which usually limit insect preference towards that plant. This type of resistant mechanism also affects certain behavioral traits of the targeted pest [30]. In this regard, numerous studies have been undertaken within the integrated pest management (IPM) framework to control pests like *A. fabae* and *A. craccivora* [31,32,33,34,35,36,37]. These efforts usually include a selection of resistant crop cultivars, exhibiting both antibiosis and antixenosis mechanisms against targeted insects [11,12,13].

Recently, Nikolova [15] reported that aphids grow and develop rapidly, enabling their populations to quickly exceed economic thresholds (10–15% of plants infested with colonies of wingless or winged individuals). Furthermore, the damage caused by numerous aphid species adversely affect plant photosynthesis, growth, and their physiological functions [38,39,40]. Moreover, the pod formation stage in *Vicia faba* is highly vulnerable against *A. fabae* in comparison to the flowering and budding stages [15]. However, we hypothesize that the susceptibility of broad beans towards the infestation of the *A. fabae* and *A. craccivora* varies at each developmental stage of the plant. Therefore, the primary objective of this study was to pinpoint the most vulnerable phenological stages of the crop plant towards the infestation of black aphids. This knowledge is crucial for safeguarding the broad bean crops during their early critical vegetative phases and mitigating both direct and indirect damages, mainly through phytovirus transmission.

## 2. Materials and Methods

### 2.1. Plant

The study was carried out in 2018, using a Histal variety of broad bean, *V. faba* L. (Fabales: Fabaceae). The Spanish origin variety (Histal) was selected due to its medium–early maturity characters and adaption to a wide range of ecological conditions, especially low temperature. The plant is usually 70–90 cm tall with 4–5 strong, thick stems. The pod is 30–33 cm long and 3 cm wide, giving on average 7–8 seeds [41]. The variety was used for both antibiosis and antixenosis tests. The beans were sown in a greenhouse at the Batna Regional Station of the National Institute for Plant Protection (NIPP). The ecological conditions, including temperature, natural photoperiod, and relative humidity, were maintained inside the greenhouse (18 ± 1 °C; L13: D11 h; and 42 ± 5% RH) [42] for both experimental mechanisms, i.e., antixenosis and antibiosis.

Seeds of the broad bean variety (Histal) were sown in plastic pots (6.5 cm diameter and 8 cm height) at alternate dates to obtain four different phenological stages of the crop (first unfolded leaf to four unfolded leaves), in order to infest them at the same time. The pots were sprinkle irrigated after every two days in order to maintain soil moisture. The weeds were removed manually.

### 2.2. Aphids

A basic breeding program for aphids was conducted, which involved wingless adults of two species, *A. craccivora* and *A. fabae*, collected from broad bean crops planted at the Batna and Biskra regions, respectively, in eastern Algeria. Aphid breeding was conducted under controlled greenhouse conditions with regulated temperature, natural photoperiod, and relative humidity levels [18 ± 1 °C; (L13: D11) h; 42 ± 5% RH] [43]. The collected aphid species were identified and verified by the expert Professor Malik Laamari, Laboratory of Agronomy, Batna 1 University, Algeria.

The first generation of aphids, obtained from collected culture, was used to infest broad bean plants for both antixenosis and antibiosis experiments. Aphids were transferred individually to each healthy plant to prevent clustering. The plants were later covered with muslin cloth to avoid the intrusion of any other insects or natural predators [44].

### 2.3. Antixenosis Test for A. craccivora and A. fabae

All plants at different phenological stages (first unfolded leaf to four unfolded leaves), were transplanted into a large circular pot measuring 25.5 cm in diameter and 25 cm in height. The plants representing distinct experimental treatments were arranged randomly in a circular formation with their leaves facing towards the center of the circle [43,45,46,47]. After forty days of initial sowing, the broad bean plants were exposed to four apterous aphids (*A. craccivora* and *A. fabae*) per crop stage. The aphids were placed in a Petri dish lid (5.7 cm diameter) and released near the circle of all plant leaves [48]. Later, the data regarding the number of aphids settled on each plant stage were recorded after 2 h, 24 h, and 48 h. Similarly, experimental protocol was followed for both insect species using fresh batches of bean plants. The experimental design followed a Randomized Complete Block Design (RCBD), with four replications.

### 2.4. Antibiosis

For an antibiosis test, two apterous adults of each aphid species were introduced separately on each bean plant, with a distinct phenological stage [49]. After producing three nymphs, the initial adults used to infest each plant were removed. Once these nymphs reached the adult stage, the females reared on the different phenological stages of bean plant were brought back to the laboratory for measuring the experimental parameter.

#### Dissection of Aphids

The female adult specimens of *A. craccivora*, which had not yet begun producing nymphs, were utilized to assess their potential fecundity. Each female was dissected under a stereomicroscope (Nikon, SMZ445, Tokyo, Japan) after being prepared with a drop of methylene blue. The ovarioles were extracted by gently pulling the terminal abdominal segment with a fine needle while securing the head and thorax with another needle. The total number of embryos and the number of mature embryos, identified by the presence of red-pigmented eyes, were counted. Embryos were classified based on size, with those displaying red eye spots being considered as developed ones [50,51,52]. The body size of adults was measured by the protocols defined by Taylor [50], which included a measurement of the specimens from the frons to the base of the cauda multiplied by the greatest width of the abdomen.

Similarly, for *A. fabae*, both body size and weight (measured with a precision scale of 0.0001 g) were determined. Linear relationships between the biological parameters of the two species were analyzed to assess the strength and significance of correlations [13]. The following parameters were evaluated: developed and total embryos of *A. craccivora*, developed embryos and adult body size of *A. craccivora*, total embryos and adult body size of *A. craccivora*, and adult body size and weight of *A. fabae*. The antibiosis tests for the two aphid species were conducted separately.

### 2.5. Statistical Analysis

Normality was assessed using the Shapiro-Wilk test adapted for small samples across all antixenosis and biological parameter tests to determine the appropriate parametric or non-parametric test. The parametric one-way ANOVA was employed for all parameters of antixenosis and antibiosis tests for both species *A. fabae* and *A. craccivora*. If significance (*p* ≤ 0.05) was found, the means of different phenological stages within each factor were compared using the Tukey post-hoc test, following verification of variance homogeneity using the Levene test. A linear correlation model was employed to investigate potential relationships using Pearson’s parametric coefficient, indicating high correlation and significance at *p* ≤ 0.05 between the biological parameters of developed and total embryos and adult body size for *A. craccivora*, as well as between the body size and weight for *A. fabae*. All statistical analyses and graphical representations were processed by SPSS statistical software version 26.0.0.0 [53].

## 3. Results

### 3.1. Antixenosis

#### 3.1.1. Test for *A. craccivora*

The four tested phenological stages of *V. faba* did not significantly (*p* > 0.05) affect the *A. craccivora* preference towards the crop plant at all tested time intervals (Table 1). The results revealed that after 2 and 24 h of the exposure period, the maximum number of adults (3.80) settled on the three unfolded leaves’ stage of crop plants, followed by the four unfolded leaf stage (3.40). A slightly lower number of adults settled on both early leaf stages of crop at both time intervals. The results further showed that after 48 h, a maximum number of adults were observed on the four unfolded leaf stage of the crop (3.20 adults), followed by three unfolded leaves (3.00 adults). However, a minimum number of adults (0.80) were observed on the first unfolded leaf stage of *V. faba* (Table 1).

#### 3.1.2. Test for *A. fabae*

The results revealed that the preference of the black bean aphid, *A. fabae,* towards *V. faba* was not significantly (*p* > 0.05) affected by the phenological stage of the crop plant at all three tested time intervals (Table 2). Overall, a slightly higher number of adults settled on the three and four unfolded leaf stage of the crop plant. The maximum number of adults after a 2 h exposure interval was recorded on the four unfolded leaf stage of the plant (3.75). Similarly, a higher number of adults was also recorded on the four unfolded leaf stage (4.75) after a 24 h exposure interval. However, after 48 h, the maximum number of adults (4.25) settled on the three unfolded leaf stage of *V. faba* (Table 2).

### 3.2. Antibiosis

#### 3.2.1. Potential Fecundity of *A. craccivora*

The current study further exhibited that the phenological stages of *V. faba* did not have a significant (*p* > 0.05) effect on the progeny production (total and developed embryos) of *A. craccivora*. The mean number of total embryos varied from 18.91 (three unfolded leaves) to 23.10 (first unfolded leaf) embryos/female. Females fed on the four unfolded leaves’ crop produced 21.50 embryos, out of which the developed ones were 11.50. The lowest developed embryos were recorded in the case of the two unfolded leaves’ crop (Table 3).

#### 3.2.2. Body Size of *A. craccivora* Adult

**Figure 1:**
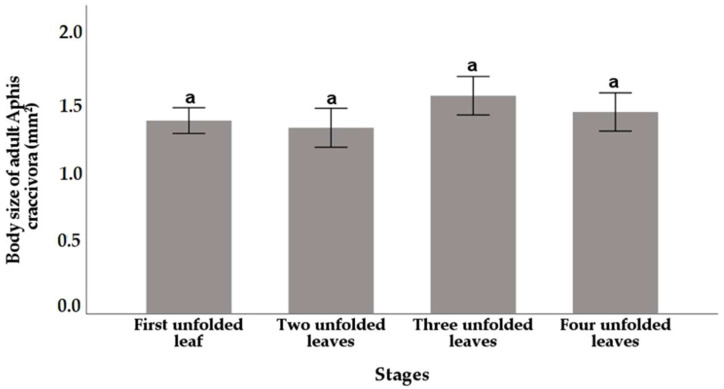
Body size (mm^2^) of *A. craccivora* adults reared on four different phenological stages of *V. faba* (mean ± SE). The bars followed by the same letters were not significantly different at (*p* ≤ 0.05). Vertical bars indicate SE.

The body size of *A. craccivora* adults did not differ significantly (*p* > 0.05) when offered leaves of different phenological stages of the *V. faba* plant (Figure 1). The adult body size was slightly larger in the case of three unfolded leaves (1.48 mm^2^), followed by four unfolded leaves (1.37 mm^2^). However, the smallest body size (1.26 mm^2^) was recorded in the case of insects, which fed on the two unfolded leaf stage of the bean plant (Figure 1).

#### 3.2.3. Body Size of *A. fabae* Adults

The results showed that the phenological stages of bean plants had a significant (*p* < 0.05) effect on the body size of *A. fabae* adults (Table 4). The maximum body size of *A. fabae* adults was recorded in the case of the first unfolded leaf stage crop (0.83 mm^2^), followed by the two unfolded leaves’ stage (0.81 mm^2^). However, adults fed on *V. faba* having three unfolded leaves had the smallest body size (0.67 mm^2^) (Table 4).

#### 3.2.4. Body Weight of *A. fabae* Adults

**Figure 2:**
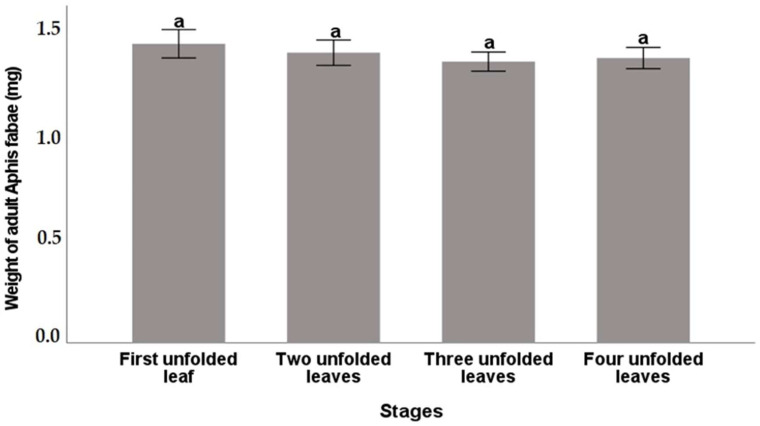
Weight (mg) of *A. fabae* adult reared on four different phenological stages of *V. faba* (mean ± SE). The bars followed by the same letters were not significantly different at (*p* ≤ 0.05). Vertical bars indicate SE.

Unlike body size, the weight of *A. fabae* adults did not differ significantly (*p* > 0.05) due to feeding on different phenological stages of bean plants (Figure 2). The results revealed that the maximum body weight of *A. fabae* adults was recorded in the case of the first unfolded leaf stage crop (1.40 mg), followed by the two unfolded leaves’ stage (1.35 mg). However, adults fed on *V. faba* having three unfolded leaves had the lowest body weight (1.31 mg) (Figure 2).

#### 3.2.5. Relationships Between Biological Parameters of *A. craccivora* and *A. fabae*

**Figure 3:**
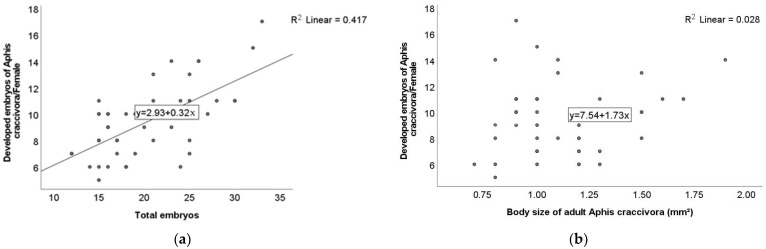

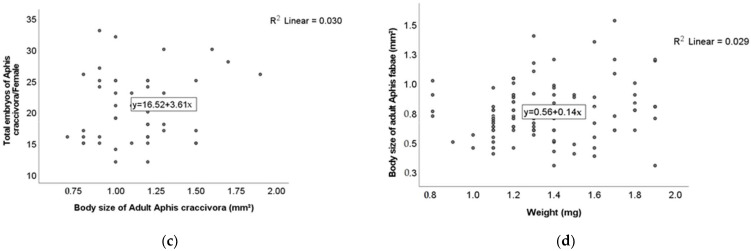
Linear correlations between number of developed and total embryos of *A. craccivora* (**a**), number of developed embryos and body size (mm^2^) of *A. craccivora* (**b**), total number of embryos and body size (mm^2^) of *A. craccivora* (**c**), body size (mm^2^) and weight (mg) of *A. fabae* (**d**).

For an *A. craccivora* adult, the results of the linear correlations showed only a positive and significant correlation (r = 0.64; *p* < 0.01) between the developed and total embryos (Figure 3a). On the other hand, the correlation test showed an absence of linear correlations for the other pairs of parameters: body size-developed embryos and body size-total embryos with a low correlation coefficient (r = 0.17; *p* > 0.05) (Figure 3b,c). The same test showed no significant relationship (r = 0.17; *p* > 0.05) between the body size and weight for an *A. fabae* adult (Figure 3d).

## 4. Discussion

Several authors had reported that plant resistance against insects is usually mediated through a mechanism of antixenosis [29,42,43,44,45,54,55]. The antixenosis leads to a variety of behaviors; some require stimulation of the sensory organs located in the aphid’s antennae, labium, and legs, while others involve movements of the head and abdomen [56]. Sensory systems such as olfaction, vision, thigmoreception, and gustation play an essential role in host selection [28]. Herbivorous insects perceive the chemicals emitted by plants to determine their precise position [57]. Storer and Van Emden [46] indicated that insects usually use visual and olfactory cues to select their hosts, whereas settling on the host requires olfactory, gustatory, and mechanical stimulation [58].

Aphids usually use different cues to determine their hosts, which mostly include color, allure, and the secondary metabollic contents of crop plants. After initial arrival, the insects take some test bites from their host plant to decide whether to stay or change their host [59]. The results regarding aphids’ attractiveness to the first four phenological stages of bean plants revealed that both aphid species, i.e., *A. craccivora* and *A fabae* exhibited enhanced attractiveness towards the three and four leaves’ stages of crop plants. Similar results were noted for both aphid species at all tested time intervals. This difference is probably explained by the physical characteristics of the oviposition site, such as the leaf, texture, color, and shape of the plant [60]. Moreover, various other factors play an important part in the choice of the host plants. Waxes on plant surfaces are complex mixtures of fatty acids, esters, and alkanes, and also contain varying amounts of various secondary metabolites. Phenological stages modify the morphology and composition of plant wax [61]. The composition of the lipids present in the epicuticles may play a role in the plants’ ability to cope with aphids. According to Powell et al. [62], chemical studies of epicuticular lipids had revealed the presence of a set of components that were wrapped up in the bean’s foliage. According to Cai et al. [63], the cultivar KOK 1679, which was resistant to the cereal aphid *Sitobion avenae*, has a high alkaloid content during the vegetative stage.

Similarly, in order to assess the effect of each stage of *V. faba* on the two-aphid species, certain biotic parameters such as the potential fecundity [64], body size [13,50], and body weight of insects were also considered [49,54,65,66,67,68]. Together, these factors fall under the umbrella of anitbiosis, referring to the negative impact of the morphological and/or chemical elements of the resistant plant on the biological functions of the arthropod pest [28]. In the current study, the highest number of developed embryos were found in the four leaves’ stage of the crop, which were statistically at par with the second leaf stage. These observed differences could probably be explained by a variation in chemical factors (secondary compounds) at the various phenological stages of the crop, which has negative effects on the aphids, leading to a reduction in their fecundity and fewer developed embryos. On the other hand, the quantity of nutrients for embryo development were insufficient during the two-leaf stage compared with the four-leaf stage. According to Huggett et al. [69], *Miscanthus sinensis* plants at the two leaves’ stage show significantly lower fecundity than the green corn aphid *Rhopalosiphum maidis* reared on plants at the five leaves’ stage.

Various researchers had demonstrated that the insect’s biological and demographic parameters, such as the size and the ability to reproduce, were directly linked to the nutritional status of the host plant. In order to protect themselves against attacks by bioaggressors, plants set up natural and artificial defenses [70]. Insect settlement, survival, growth and development, as well as fecundity can be influenced by these defenses [71]. Antibiosis is a method of protecting plants against insect colonization, where they impact insect growth, survival, or reproduction using chemical or morphological elements [28,72].

In the current study, the adult body size of *A. craccivora* was slightly larger in the case of three unfolded leaves. Furthermore, the maximum body size of *A. fabae* adults was recorded in the case of the first unfolded leaf stage crop. These results probably confirm the progressive production of toxic substances during the development of the bean plant. Moreover, several authors, such as Brnays and Chapman [61] and Strebler [73], had demonstrated that the insect’s biological and demographic characteristics, such as its size and potential fecundity, were directly linked to the nutritional status of the host plant. The results reported that the weight parameter of adult *A. fabae* did not differ statistically between the four tested phenological stages of bean plants. The study of linear correlations of the biological parameters of *A. craccivora* showed only one positive relationship between the number of developed embryos and total embryos. Similar findings had been reported by Meradsi and Laamari [13] and Meradsi [49] for *A. fabae*. Linear correlation analysis showed no relationship between the body size and weight of *A. fabae* adults.

## 5. Conclusions

The findings suggest that the early stages of broad bean are more resistant to black aphid’s attack than the later leaf stages. Although the impact between different leaf stages was non-significant, the early leaf stages might be less attractive due to less surface area and less nutrient contents. Aphids, preferably at the reproduction stage, need a healthy diet; therefore, host plants are selected according to the nutritional content of the leaf and its surface area to avoid overcrowding. Therefore, it is recommended to implement plant protection measures during the sensitive stages of broad bean development for maximum protection against aphid colonization. The control measure usually becomes less effective when the pest population reaches its ETL limit.

## Figures and Tables

**Table 1:** *Aphis craccivora* adults settled on four different phenological stages of *V. faba* (mean ± SE).

Phenological Stages	2 h	24 h	48 h
First unfolded leaf	0.60 ± 0.6 ^a^	0.40 ± 0.24 ^a^	0.80 ± 0.37 ^a^
Two unfolded leaves	3.00 ± 0.89 ^a^	2.80 ± 0.73 ^a^	2.20 ± 0.73 ^a^
Three unfolded leaves	3.80 ± 0.66 ^a^	3.80 ± 1.15 ^a^	3.00 ± 0.89 ^a^
Four unfolded leaves	3.40 ± 1.2 ^a^	3.40 ± 1.2 ^a^	3.20 ± 1.24 ^a^
*p*	0.080 ^ns^	0.078 ^ns^	0.235 ^ns^
F	2.702	2.729	1.572
Df	3/12	3/12	3/12

^ns^: not significant; the means followed by the same letters in the same column were not significantly different at *p* ≤ 0.05.

**Table 2:** *Aphis fabae* adults settled on four different phenological stages of *V. faba* (mean ± SE).

Phenological Stages	2 h	24 h	48 h
First unfolded leaf	3.25 ± 1.65 ^a^	2.50 ± 1.19 ^a^	3.00 ± 1.22 ^a^
Two unfolded leaves	2.75 ± 1.03 ^a^	2.75 ± 1.37 ^a^	3.00 ± 1.22 ^a^
Three unfolded leaves	2.75 ± 0.85 ^a^	3.00 ± 1.22 ^a^	4.25 ± 1.65 ^a^
Four unfolded leaves	3.75 ± 0.75 ^a^	4.75 ± 1.31 ^a^	3.50 ± 1.19 ^a^
*p*	0.908 ^ns^	0.605 ^ns^	0.898 ^ns^
F	0.180	0.637	0.195
Df	3	3	3

^ns^: not significant; the means followed by the same letters in the same column were not significantly different at *p* ≤ 0.05.

**Table 3:** Total embryos and developed embryos of *A. craccivora* females fed on four phenological stages of *V. faba* (mean ± SE).

Phenological Stages	Total Embryos	Developed Embryos
First unfolded leaf	23.10 ± 1.47 ^a^	10.50 ± 0.68 ^a^
Two unfolded leaves	19.30 ± 1.37 ^a^	8.70 ± 0.68 ^a^
Three unfolded leaves	18.91 ± 1.46 ^a^	9.18 ± 0.60 ^a^
Four unfolded leaves	21.50 ± 2.83 ^a^	11.50 ± 1.05 ^a^
*p*	0.230 ^ns^	0.062 ^ns^
F	1.51	2.687
df	3	3

^ns^: not significant; the means followed by the same letters in the same column were not significantly different at *p* ≤ 0.05.

**Table 4:** Body size (mm^2^) of *A. fabae* adults reared on four different phenological stages of *V. faba* (mean ± SE).

Phenological Stages	Body Size (mm^2^)
First unfolded leaf	0.83 ± 0.04 ^a^
Two unfolded leaves	0.81 ± 0.05 ^a^
Three unfolded leaves	0.67 ± 0.04 ^b^
Four unfolded leaves	0.70 ± 0.03 ^ab^
*p*	0.03 *
F	2.997
Df	3

*: significant at *p* ≤ 0.05; the means followed by the different letters in the same column were significantly different at *p* ≤ 0.05.

## Data Availability

The original contributions presented in this study are included in the article. Further inquiries can be directed to the corresponding authors.
